# Aureochrome 1 Illuminated: Structural Changes of a Transcription Factor Probed by Molecular Spectroscopy

**DOI:** 10.1371/journal.pone.0103307

**Published:** 2014-07-24

**Authors:** Silke Kerruth, Kenichi Ataka, Daniel Frey, Ilme Schlichting, Joachim Heberle

**Affiliations:** 1 Experimental Molecular Biophysics, Freie Universität Berlin, Berlin, Germany; 2 Biomolecular Mechanisms, Max Planck Institut for Medical Research, Heidelberg, Germany; Julius-Maximilians-University Würzburg, Germany

## Abstract

Aureochrome 1 from *Vaucheria frigida* is a recently identified blue-light receptor that acts as a transcription factor. The protein comprises a photosensitive light-, oxygen- and voltage-sensitive (LOV) domain and a basic zipper (bZIP) domain that binds DNA rendering aureochrome 1 a prospective optogenetic tool. Here, we studied the photoreaction of full-length aureochrome 1 by molecular spectroscopy. The kinetics of the decay of the red-shifted triplet state and the blue-shifted signaling state were determined by time-resolved UV/Vis spectroscopy. It is shown that the presence of the bZIP domain further prolongs the lifetime of the LOV_390_ signaling state in comparison to the isolated LOV domain whereas bound DNA does not influence the photocycle kinetics. The light-dark Fourier transform infrared (FTIR) difference spectrum shows the characteristic features of the flavin mononucleotide chromophore except that the S-H stretching vibration of cysteine 254, which is involved in the formation of the thio-adduct state, is significantly shifted to lower frequencies compared to other LOV domains. The presence of the target DNA influences the light-induced FTIR difference spectrum of aureochrome 1. Vibrational bands that can be assigned to arginine and lysine side chains as well to the phosphate backbone, indicate crucial changes in interactions between transcription factor and DNA.

## Introduction

Blue-light (BL) photoreceptors play crucial roles in plant development and phototropism. The recently discovered BL receptor aureochrome (Aureo) from *Vaucheria frigida* controls the light dependent development of these Xanthophytes. *Vaucheria frigida* owns two different aureochromes, aureochrome 1 and aureochrome 2. While aureochrome 1 mediates the BL-induced branching process of the cell/filaments [1], aureochrome 2 controls the development of a sex organ. Both aureochromes contain a LOV (light-, oxygen- and voltage-sensitive) domain and a basic region/leucine zipper (bZIP) domain [2].

LOV domains, a subfamily of the Per-ARNT-Sim (PAS) family, are common BL sensitive domains, present in many light-sensitive proteins [3]. These domains show a typical PAS domain fold consisting of a five-stranded antiparallel β-sheet and four helices. A non-covalently bound flavin mononucleotide (FMN) moiety located within this fold acts as the chromophore of the domain [4]. The ground state (LOV_447_) of LOV domains has an absorption maximum at around 447 nm. When a blue photon is absorbed by the FMN chromophore, the protein undergoes a photocycle that includes two spectrally distinct intermediate states. The first intermediate LOV_715_ is formed on a nano-second timescale due to intersystem crossing from the exited FMN to the triplet state. The characteristic absorption maxima are at 650 and 715 nm [5]. Subsequently the triplet state decays through a neutral radical state [6] into the LOV_390_ intermediate which is characterized by a covalent bond between the C_4a_ of the isoalloxazine ring of FMN and a nearby cysteine residue and an absorption maximum at 390 nm [5], [7]. Depending on the LOV domain, the lifetime of the covalent adduct ranges from few seconds to several minutes [7]–[10].

**Figure 1:**
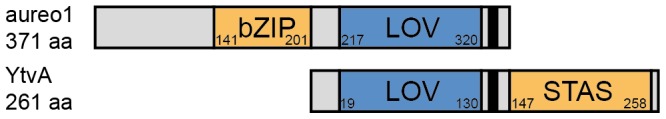
Schematic domain drawing of aureochrome 1 and YtvA. Domain organization of full-length aureochrome 1 from *Vaucheria frigida* and of YtvA from *Bacillus subtilis*. The LOV domains are coloured in blue, while the effector domains are colored in orange. The J_α_-helix is located C-terminal to the LOV domains (shown as a black bar).

Typically, LOV domains are connected by a C-terminal α-helix, the so called J_α_-helix, to a downstream effector domain (Fig.1). The effector domains can have various functions, e.g. kinase activity, sulfate transporter or transcription factor [11], [12]. In contrast, the J_α_-helix is not the linker between the two domains of aureochromes [13] as the effector domain, here a bZIP domain, is located at the N-terminus. bZIP domains consist of a basic region that is responsible for DNA recognition and a leucine zipper helix. This leucine zipper comprises a leucine residue at each seventh amino acid (minimum three of a kind) and can form a coiled-coil structure [14], [15]. It was shown that aureochrome 1 recognizes the sequence TGACGT and, therefore, was suggested to belong to the class of S-type bZIP domains [2], [16], [17].

The combination of a light-sensitive domain with a DNA binding domain makes aureochrome 1 of particular interest for the field of optogenetics due to the possibility of controlling gene expression by light. However, the functional mechanism of signal transfer from the blue-light absorbing LOV domain to the downstream bZIP domain has not been explored, yet. In phototropin and YtvA the J_α_-helix, which is a conserved structure element between the LOV and the effector domain, plays a crucial role. Thus, FTIR studies showed that the helix unfold upon illumination, leading to kinase activation [18], [19]. In contrast, STAS domain activation in YtvA probably takes place via light-induced dimerization as shown by SEC and CD [20], [21]. In fact, dimerization and structural changes like α-helical unfolding also seem to play an essential role in the signal transfer of aureochrome 1 as recently shown [22], [23]. However, the blue-light activated dimerization is still under debate. While Herman et al. and Toyooka et al. claim the J_α_-helix to be mandatory for light-induced dimerization of the isolated LOV domain [16], [23], Hisatomi et al. observed only monomers of this construct, irrespective of illumination [22]. However, all studies agree on the presence of dimers independent of illumination as soon as longer constructs are used, most probably due to disulfide bond formation [22]. These results give evidence that light induced dimerization is not the activation step for DNA binding. Furthermore, it is discussed, also for other LOV domains like VVD, that the N-terminal located α-helical cap can replace the J_α_-helix in its function [24].

Here, we describe studies of the photoreaction of full-length aureochrome 1 by time-resolved UV/Vis spectroscopy. The kinetics of the decay of the triplet state as well as of the adduct state were determined. The full-length aureochrome 1 shows a longer life time of the signaling state compared to the isolated LOV domain. Furthermore, light-induced FTIR difference spectroscopy was used to elucidate the influence of binding the target DNA to aureochrome 1 on the photocycle intermediate LOV_390_. We were able to demonstrate interactions of arginine and lysine side chains of aureochrome 1 with the phosphate group of the DNA backbone. Based on homology we propose formation of a Y shaped DNA-aureochrome 1 complex, where the DNA is bound in between the two basic regions of the bZIP domain in the major grove.

## Material and Methods

### Protein Expression and Purification

A codon optimized synthetic construct (GenScript) of full length aureochrome1 (Uniprot ID: A8QW55) was cloned into pET28a (Novagen) containing an N-terminal 6xHis-tag and a Thrombin cleavage site. One plate of freshly transformed *E.coli* BL21(DE3) cells were used to inoculate the main culture consisting of 2 l LB media. Cells were grown in the dark at 37°C to an OD_600nm_ of 0.6. The temperature was changed to 20°C and protein expression was induced using 0.5 mM IPTG. After 18 h cells were harvested by centrifugation (4°C, 30 min 3000*g), flash frozen in liquid nitrogen and stored at −80°C.

Cells of a 2 l expression culture were suspended in 50 ml buffer A (50 mM Na_2_HPO_4_/NaH_2_PO_4_, 300 mM NaCl, 10 mM imidazole, 2 mM β-mercaptoethanol, pH 8.0) containing DNase, lysozyme (2 mg/ml, final concentration) and protease inhibitors. The solution was stirred for 1 h in the dark at 4°C and cells were disrupted by a microfluidizer (Microfluidics). Unbroken cells and debris were removed by centrifugation (SS-34, 4°C, 16000 rpm, 1 h). The supernatant was loaded to 3.5 ml Ni-NTA beads (Qiagen) equilibrated in buffer A. The column was washed with 40 ml buffer A followed by 40 ml buffer A supplemented with 29.6 mM imidazole. Protein was eluted with 10 ml buffer B (50 mM Na_2_HPO_4_/NaH_2_PO_4_, 300 mM NaCl, 500 mM imidazole, 2 mM β-mercaptoethanol, pH 8.0). Directly after elution β-mercaptoethanol was added to a final concentration of 10 mM and the protein was dialyzed against 2×500 ml buffer C (10 mM Na_2_HPO_4_/NaH_2_PO_4_, 200 mM NaCl, 2 mM EDTA, 10% (v/v) glycerol, 10 mM β-mercaptoethanol, pH 8.0). The dialyzed sample was loaded on a 5 ml Heparin HiTrap column (GE Healthcare) equilibrated with buffer D (10 mM Na_2_HPO_4_/NaH_2_PO_4_, 100 mM NaCl, 10% (v/v) glycerol, 10 mM β-mercaptoethanol, pH 8.0). Protein was eluted using a liner gradient to 1 M NaCl in buffer D. Fractions containing aureochrome 1 were concentrated (30 kDa cutoff (Amicon)) and further purified using a Superose 6 column (23 ml, GE Healthcare) equilibrated in buffer E (10 mM Na_2_HPO_4_/NaH_2_PO_4_, 100 mM NaCl, 10% (v/v) glycerol, 10 mM DTE, pH 8.0). The pure protein with a ratio of A_280_/A_450_ = 2.8 was concentrated to 10 mg/ml, flash frozen in liquid nitrogen and stored at −80°C.

### Time-resolved UV/VIS absorption spectroscopy

The sample of aureochrome 1 was diluted to a concentration of 20 µM in buffer F (10 mM Na_2_HPO_4_/NaH_2_PO_4_, 200 mM NaCl, 10 mM DTE, 10% glycerol, pH 8, 20°C). Concentrations of the samples were determined by the absorption at 450 nm with an estimated extinction coefficient of FMN of about 12500 M^−1^·cm^−1^. The sample was illuminated for 5 s with a light emitting diode (LED from Luxeon Star, emission maximum at 455 nm and 30 nm FWHM, power density of 10 mW/cm^2^) while the spectrum was recorded from 250–700 nm using a UV/Vis spectrometer (SHIMAZU UV-2450). After switching-off the LED, spectra were collected at 45 s intervals. Up to five kinetic experiments were averaged to improve the signal-to-noise ratio of the spectra.

For measurements in the presence of DNA, oligomers with the target sequence 5′-TCTGACGTGA-3′ and 5′-TCACGTCAGA-3′, purchased from Eurofins MWG (HPLC purified), were used. The reverse and forward single-strand oligomer were mixed, concentration each 10 mg/ml, and incubated for 10 minutes on ice. This double-stranded DNA (dsDNA) was added to aureochrome 1 solution in stoichiometric amounts and incubated for 1 hour on ice.

### Flash photolysis experiments

For UV/Vis experiments with ns time resolution, 59 µM aureochrome 1 in buffer F was excited by a 10 ns laser pulse at 475 nm from a solid-state laser (Nd:YAG driven OPO, energy density of 5 mJ/cm^2^, one pulse every 30 minutes). The measuring light was the emission from a halogen bulb passing a monochromator set to the desired wavelength. The time-dependent absorption changes of the sample were detected by a photomultiplier tube and recorded by two separate transient recorders with digitization rates of 100 MHz and 2 MHz, respectively. Data were averaged on a quasi-logarithmic time-scale and merged to yield time traces covering the time range from 10 ns to 1 s. Three kinetic traces were averaged at each wavelength selected (390, 660 and 715 nm).

### FTIR spectroscopy

Light-induced FTIR difference spectroscopy was performed as previously described [25]. Briefly, 10 µL of the sample (330 µM) in buffer F was concentrated on a BaF_2_ window using a gentle stream of dry air. After drying, the sample was still well hydrated (amide I/amide II ratio 2∶1). Sample excitation was performed by the same LED as for the UV/Vis experiments (see above). Infrared experiments were performed on an IFS 66v/S spectrometer (Bruker Optics, Ettlingen, Germany). FTIR difference spectra were calculated by subtraction of the dark state spectra from the spectra recorded under photo-stationary conditions. Spectra were recorded at a spectral resolution of 4 cm^−1^ and represent the average of 100,000 scans.

## Results

### Kinetics of the aureochrome 1 photocycle

**Figure 2:**
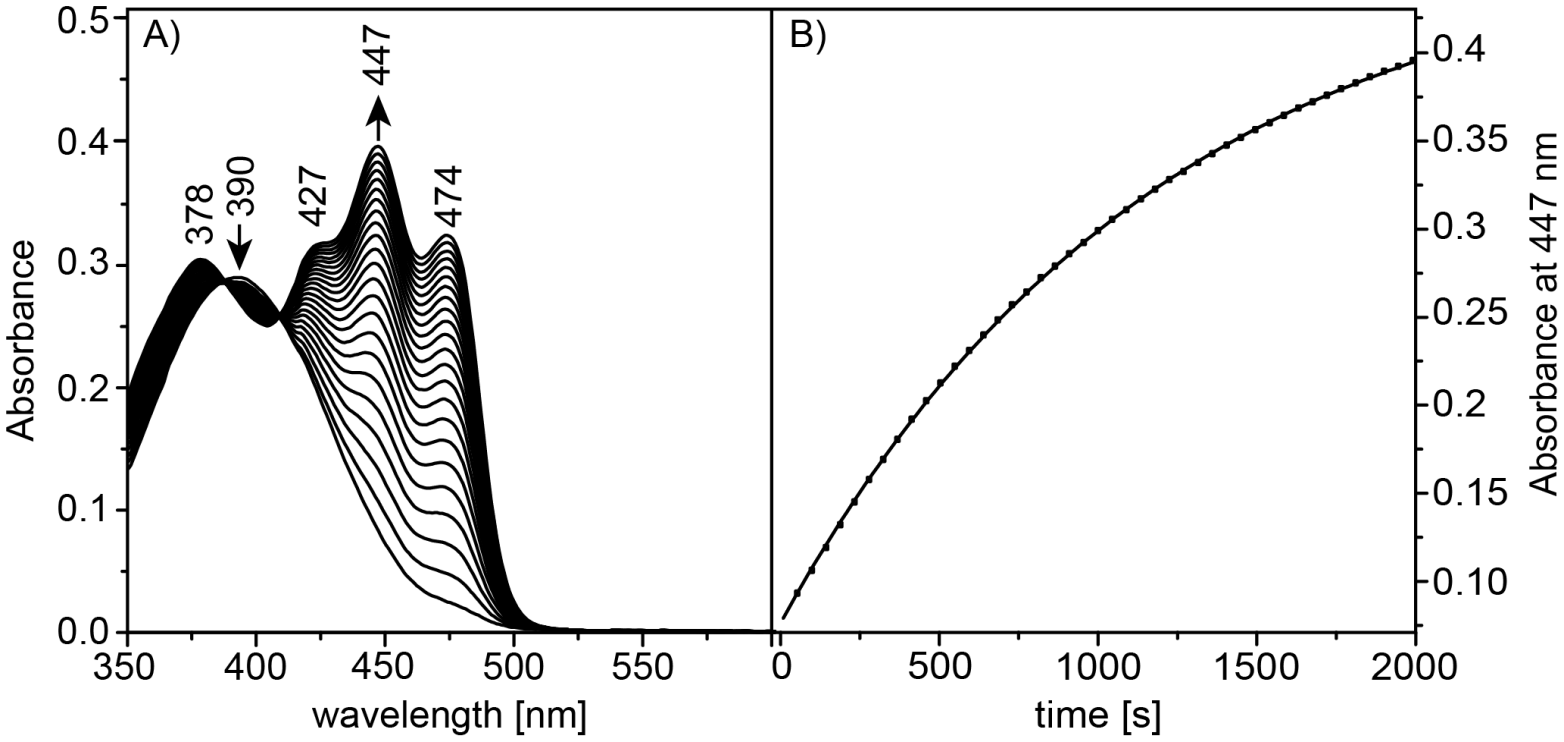
Kinetic of the ground state recovery. A) Absorption spectra of aureochrome 1 recorded during the recovery of the dark state. The sample was illuminated with blue-light and spectra were taken at intervals of 45 s. The arrows indicate the increase and decrease of the maxima of the LOV_445_ and LOV_390_ states, respectively. B) Kinetics of recovery of the absorbance at 446 nm of aureochrome 1 after 5 s blue light illumination. The continuous line represents a single exponential fit to the data (dots). The time constant for the ground state recovery was determined to 22±1 min.

The absorption spectrum of aureochrome 1 exhibits two major absorption bands, in the UVA (380 and 315 nm) and in the blue region (410–490 nm, Fig.2A). The long wavelength absorption at 447 nm shows the typical vibronic fine structure of oxidized FMN with side maxima at 425 nm and 474 nm, when harbored in the peptide environment of a LOV domain. Like the LOV2 domain from plant-type phototropin [4], aureochrome 1 lacks the double-peak structure and shows only one band with a maximum at 378 nm.

Upon absorption of a blue photon, the band feature at around 450 nm is bleached. Bleaching is fully reversible when illumination is switched off and the spectrum of the dark state is recovered in about 45 min. Isosbestic points at 387 and 409 nm indicate that the recovery reaction represents a transition between two states (Fig. 2A). Absorption changes were followed at 446 nm to determine the kinetics of the recovery reaction of the initial dark state. Experiments were carried out in the absence and presence of a 10-bp double-stranded DNA oligomer which carried the binding motif of the aureochrome 1 bZIP domain. For both samples, the plot of the absorption at 446 nm versus time indicates a mono-exponential recovery of the ground state (Fig. 2B). The time constant is 22±1 min (at 20°C) and is not influenced by the presence of the target DNA oligomer (data not shown).

**Figure 3:**
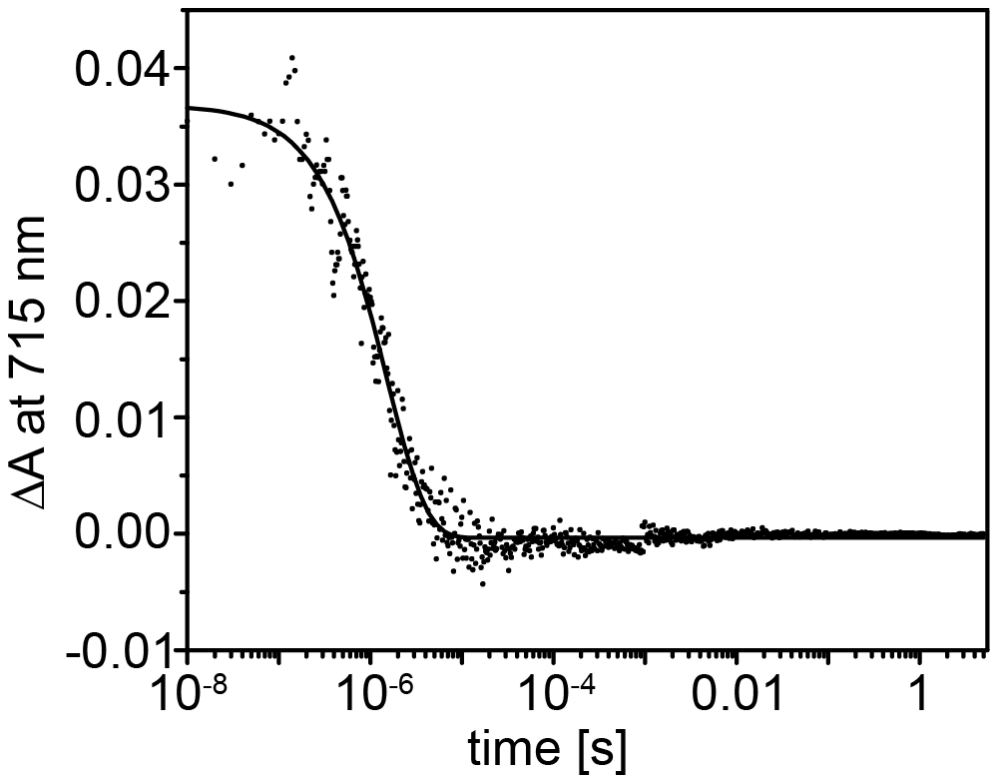
Kinetic of the triplet state decay. Kinetics of the absorbance changes at 715(absorption band of the triplet state) of aureochrome 1 after a 10 ns laser flash. Three recordings with a time delay of 30 min after each laser flash have been averaged. The continuous line is the single exponential fit to the data yielding a time constant of τ = 1.5±0.1 µs.

After blue-light absorption, aureochrome 1 undergoes a photocycle in which the first intermediate is formed within a few nanoseconds. This intermediate exhibits a massively red-shifted absorption maximum characteristic for the triplet state of the FMN cofactor [5]. We monitored the decay of the triplet state by recording absorption changes at 715 nm with a time resolution of 50 ns after pulsed laser excitation (Fig. 3). A single exponential decay was observed and the fit yielded a time constant of 1.4±0.2 µs. Again, addition of the target DNA to aureochrome 1 resulted in identical kinetics (data not shown).

### Response of the LOV domain

Structural changes occurring upon conversion of the dark state to the blue-shifted intermediate state (LOV_390_) were monitored by vibrational spectroscopy. As the lifetime of the LOV_390_ intermediate is very long (*vide supra*), the vibrational changes can be recorded under photo-stationary conditions without the need for time-resolved experimentation. Positive bands in the light-dark FTIR difference spectra correspond to the long-lived adduct intermediate LOV_390_ and negative bands to vibrations of dark-state LOV_447_. Most of the difference bands are due to vibrations of the chromophore because FMN undergoes the largest dipolar changes of the holoprotein [25].

**Figure 4:**
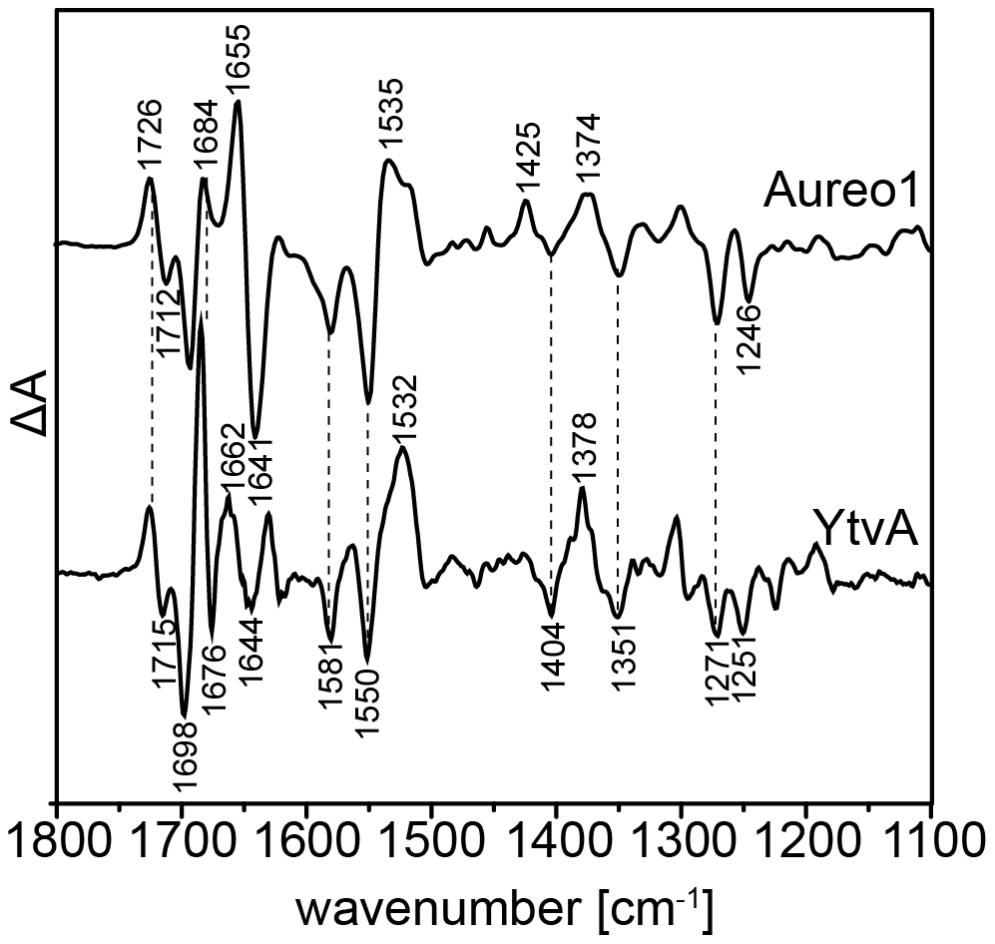
FTIR difference spectrum of aureochrome 1 in the region of 1800 to 1100^−1^. FTIR difference spectrum (light-dark) of aureochrome 1 (top spectrum) recorded under continuous illumination with blue-light (light emitting diode with λ_max_ = 455 nm). For comparison, the difference spectrum of YtvA (from *Bacillus subtilis*) is also shown (bottom spectrum, re-plotted from [26]). The numbers indicate the frequencies of vibrational bands discussed in the text and the vertical dashed lines point to bands that are identical in both spectra.

Band assignment is facilitated by the comparison to other LOV domain proteins. For this purpose, FTIR differences of full-length YtvA recorded under identical conditions [26] are included in Figure 4 (lower trace). YtvA from *Bacillus subtilis* comprises a LOV domain and a downstream STAS domain, which is involved in general stress response of this bacterium [27]–[29]. The sequence identity between both LOV domain is 52%. As expected, the two spectra share similarities but detailed inspection reveals crucial differences. Particularly differing features are observed in the amide I (1690–1620 cm^−1^) and amide II (1570–1520 cm^−1^) regions indicating changes in secondary structure. The negative band at 1676 cm^−1^ of dark-state YtvA is absent in the spectrum of aureochrome 1 and the intensity of the positive peak at 1684 cm^−1^ is decreased. In contrast, the negative band at 1641 cm^−1^, which is assigned to the ν(C = C) vibration of ring I of the isoalloxazine moiety [25], is more intense than in the YtvA spectrum. We suspect contributions from changes in the amide I band, which are indicative for changes in the secondary structure of aureochrome 1, and overlap with the chromophore mode. Furthermore, the stretching vibration of C_4_ = O_4_ oscillates with a frequency of 1712 cm^−1^, the in-plane bending vibration of N_3_-H at 1374 cm^−1^ and the ring vibration involving mainly ν(C_4_-N_3_), ν(C_2_-N_3_), ν(C_4_-C_4a_), ν(C_2_-N_1_), δ(C_2_ = O_2_) at 1246 cm^−1^. In aureochrome 1, the atoms O_4_, N_3_ and O_2_ of ring III of the FMN form hydrogen bonds with the side chains of N286, N296 and Q317, residues that are highly conserved in LOV domains [13]. Thus, the vibrations of ring III are influenced by the strength of the hydrogen bonds. The observed shift by 3 to 5 cm^−1^ to lower wavenumbers corresponds to an increase of the strength of the hydrogen bond network formed with the chromophore.

**Figure 5:**
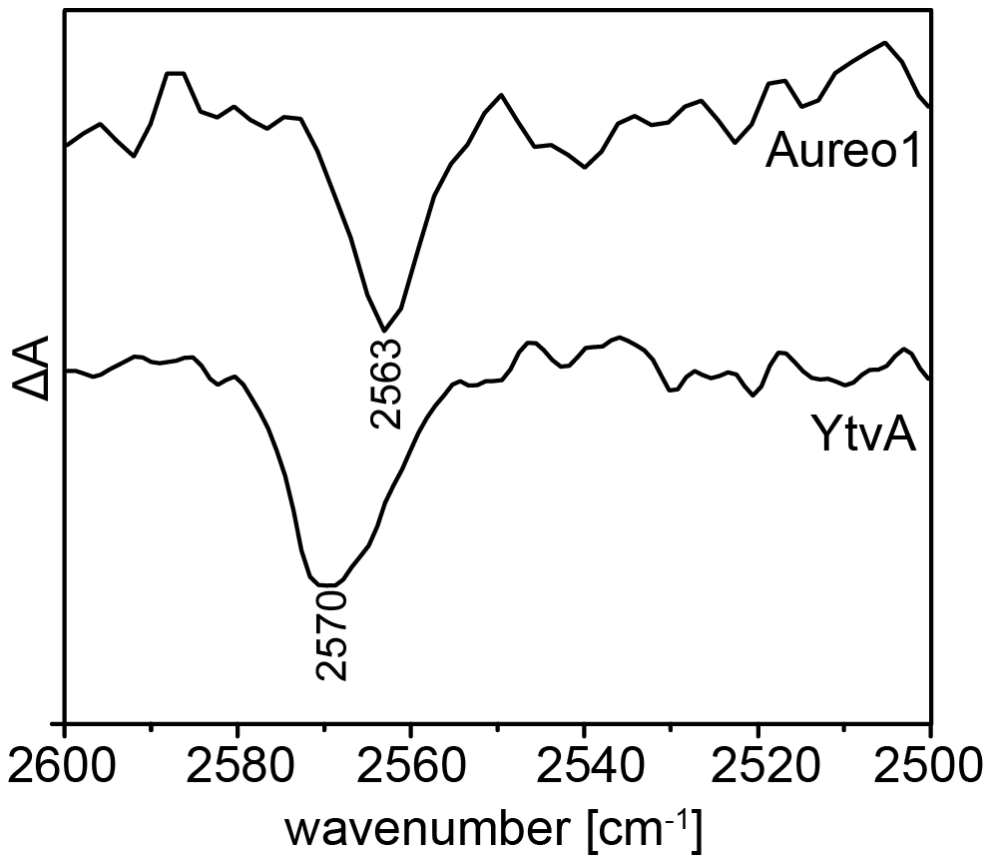
FTIR difference spectrum of aureochrome 1 in the region of 2500 to 2600^−1^. Light-dark difference spectra of aureochrome 1 (upper line) and full-length YtvA (lower line) from *Bacillus Subtilis* in the region between 2500 and 2600 cm^−1^ [26]. The negative bands correspond to the S-H stretching vibration of a cysteine in the dark state which is lost upon formation of the adduct state (C254 in aureochrome 1 and C62 in YtvA).

The adduct state is characterized by a covalent bond between the C_4a_ of the isoalloxazine ring of FMN and the sulfur of a nearby cysteine. Since the cysteine is protonated in the ground state, the S-H stretching vibration appears as a negative band in the FTIR difference spectrum. In fact, a negative band at 2563 cm^−1^ is observed and, thus, assigned to C254 of the ground state of aureochrome 1 (Fig.5). The frequency of the S-H stretching vibration of C254 is downshifted by 7 cm^−1^ in comparison to the corresponding vibrations in YtvA, LOV1 and LOV2 of phototropin at 2570 cm^−1^ [26], The position of this band rather fits to a shoulder at 2562 cm^−1^ that was observed in the spectra of LOV1 domain. The appearance of this shoulder was interpreted as the band of the second rotamer configuration of the side chain of cysteine. One rotamer is closer to FMN (distance S to N_5_ is 3.5 Å instead of 3.9 Å [30]) and in a more polar environment as the other rotamer, which is in close vicinity (distance 3.3 Å) to the methyl group of a nearby leucine residue (L257 in aureochrome 1). This interpretation is in line with the frequency shift of the S-H stretching vibration to lower wavenumbers when organic thiols are dissolved in polar solvents [31]. Therefore, we infer from our IR study that C254 of aureochrome 1 seems to prefer the rotamer configuration that is closer to N_5_ of the isoalloxazine ring of FMN.

### DNA target binding

**Figure 6:**
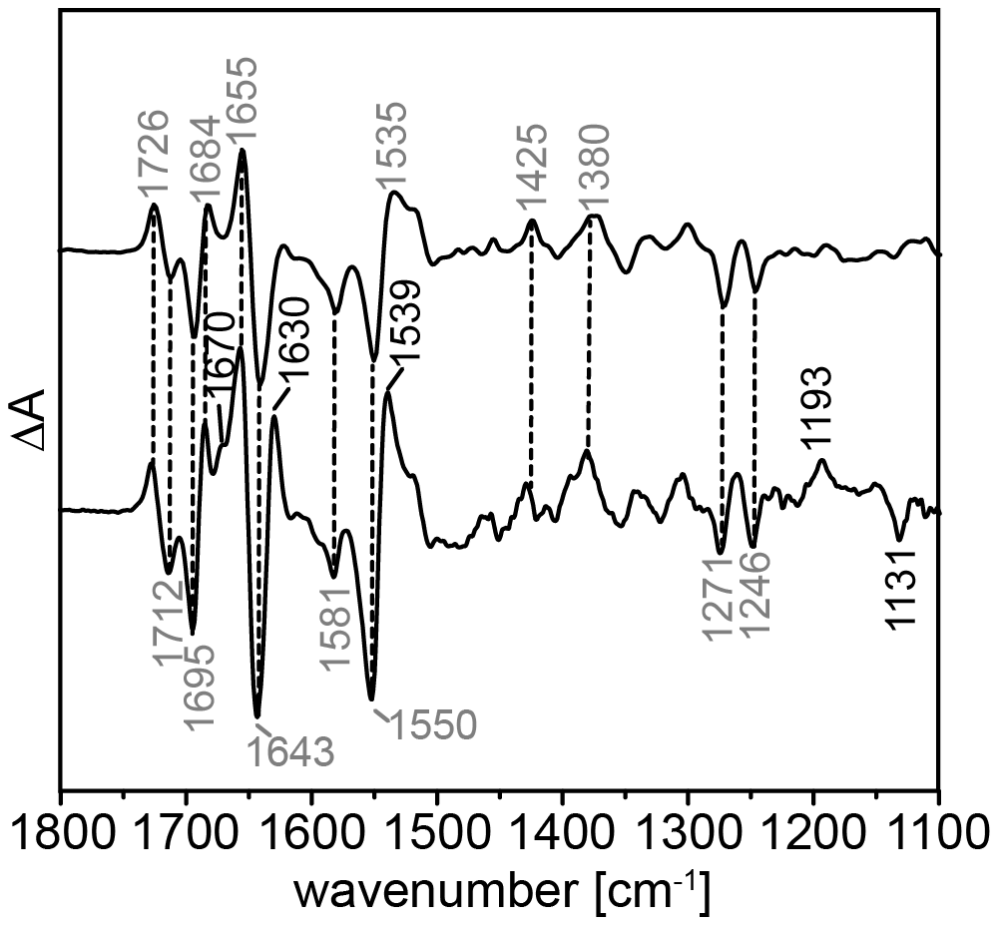
FTIR difference spectrum of aureochrome 1 (1800–1100 cm^−1^) with bound DNA. Light-dark FTIR difference spectra of aureochrome 1 in the absence (top) and presence (bottom) of dsDNA oligomer (10 bp) with the target sequence TGACGT. The numbers indicate the position of the positive and negative bands and the vertical dashed lines identify bands with same position in both spectra. Bands labeled by black numbers indicate new bands that appear due to the target DNA binding. Grey labeled bands are invariant to the presence of DNA.

In addition to the light-absorbing LOV domain, aureochrome 1 contains a bZIP domain which binds the target DNA. We studied the effect of the substrate DNA by recording IR difference spectra of aureochrome 1 in the absence (upper trace in Figure 6) and in the presence (lower trace) of its target DNA. The difference spectra share the typical FMN bands but some signals are significantly increased. Bands at 1684 and 1655 cm^−1^ almost double their intensities and a shoulder at 1670 cm^−1^ appears in the difference spectrum in the presence of DNA. Beside the amide I vibrations that are sensitive to structural changes, the C_4_ = O_4_ stretching vibration of thymine bases (1671–1655 cm^−1^ for T, ds) and the C_6_ = O_6_ stretching vibration of guanine bases (1678–1689 cm^−1^ for G, ds) appear in this frequency range [32]. In addition to the shoulder at 1670 cm^−1^, a positive band rises at 1630 cm^−1^. These two vibrational bands absorb in the region of the asymmetric and symmetric vibrations of arginine side chains, respectively [33], [34]. A positive band appears at 1539 cm^−1^ which may be indicative for the symmetric N-H deformation vibration of the terminal amino groups of lysine side chains (about 1530 cm^−1^) [33]. The asymmetric deformation vibration of the NH_3_^+^ group lies at around 1629 cm^−1^ and probably overlaps with the symmetric one of the arginine side chain [35]. In the phosphate/sugar region, two bands at 1193 (pos) and 1131 (neg) cm^−1^ appear due to DNA addition. In this region PO_2_ stretching vibrations of the DNA backbone occur. The frequency of 1193 cm^−1^ corresponds to P = O stretch of a POOH group, while at 1131 cm^−1^ P-O stretching vibrations with a single bond character are located.

## Discussion

The photoreaction of full-length aureochrome 1 from *Vaucheria frigida* was studied by molecular spectroscopy. The visible absorption spectrum shows only one broad band in the UVA region, which indicates that the LOV domain of aureochrome1 resembles the LOV2 domain of phototropin [4] and LOV domain of YtvA [36]. This is in line with the fact that T222 and N229 in aureochrome 1, which strongly influence the spectral features in the UVA range [37] are conserved in LOV2 domains. Time-resolved UV/Vis measurements were performed to determine the kinetics of the photocycle intermediates. The decay of the LOV_715_ triplet state is characterized by a time constant of 1.4±0.2 µs. This value agrees fairly well with the time constant of 2.8 µs as derived by light-induced transient-grating (TG) spectroscopy [16] and is in the range of other LOV domains like phototropin1 LOV1 (4 µs), LOV2 (1.9 µs) and YtvA (2 µs) [7], [9], [10]. Unfortunately, it was impossible to record the rise kinetics of the LOV_390_ state due to the triplet state LOV_715_ absorption in this region that cancels the former changes [5].

The decay of LOV_390_ proceeds in full-length aureochrome 1 with a time constant of 22 min. This is significantly longer than the lifetimes of the isolated AuLOV domain, irrespective if the J_α_-helix is absent (4.9 min) [2] or present (8 min) [2], [13]. Thus, we conclude that the presence of the bZIP domain prolongs the lifetime of the signaling state LOV_390_. Studies of constructs of aureochrome1a from *Phaeodactylum tricornutum* reported an increase of the decay constant from 6 to 38 min due to the presence of the J_α_-helix [23]. However, experiments on the full-length protein were not reported.

The light-dark FTIR difference spectrum exhibited the typical bands of the FMN chromophore in the range of 1000 to 1800 cm^−1^. However, the ring III vibrations of the FMN show a shift to lower wavenumbers in comparison to YtvA, which is an indication for a stronger hydrogen-bonding to the oxygen of the carbonyls of ring III. The H-bonded network is comprised of residues N286, N296 and Q317. Such minute structural differences are usually not resolved in the crystal structures.

The light-induced FTIR difference spectrum shows a negative band at 2563 cm^−1^ that corresponds to the S-H stretching vibration of C254 in dark-state aureochrome 1. The downshift of this band by 7 cm^−1^ in comparison to other LOV domains indicates a more polar environment of the S-H group compared to other LOV domains. We infer that the conformer where the S-H is closer to the N_5_ of the isoalloxazine ring, is the dominant conformer in aureochrome 1 (see Bednarz et al. for a discussion [26]). The high resolution structures of Cr-phot-LOV1 and YtvA show two rotamer configurations for the corresponding cysteine with a closer distance of 3.5 Å to the N_5_ and 3.3 Å to the terminal methyl group of a close-by leucine [21], [30]. The crystal structure of Aureo1-LOV was determined at 2.8 Å resolution [13], which does not allow for the identification of alternative configurations. The rotamer configuration of C254 identified by Mitra et al. corresponds to the rotamer pointing towards L257 with a distance of the sulfur to N_5_ of the isoalloxazine ring of 3.8 Å [13].

Upstream of the LOV domain, aureochrome 1 harbors a bZIP domain. The latter is known to bind DNA after dimerization of the leucine zipper domain with its basic region at the major grove. By formation of a coiled coil of two helices, a Y shape structure is created that can bind the target DNA by specific interactions of the C-terminal basic region with the DNA, mostly via hydrogen bonds (see Fig.6 left).

The interaction of DNA and the bZIP domain is reflected in the FTIR difference spectrum (see Fig.6, lower trace). We observe a negative band at 1131 cm^−1^ that is assigned to the P-O stretching vibration with single bond character [38]. Formation of a hydrogen bond to one of the phosphate oxygens leads to strengthening of the double bond character of the other P-O bond. As a consequence, the P = O stretching vibration with a double bond character shows up at 1193 cm^−1^ [38]. Furthermore, asymmetric and symmetric C = N stretching vibrations assigned to arginine side chains are observed at 1630 and 1670 cm^−1^. At the present stage, we are not able to assign the vibrations to specific arginine residues due to the lack of proper point mutants. Additionally, peaks at 1539 and 1630 cm^−1^ corresponding to the asymmetric and symmetric deformation vibration of the NH_3_^+^ group, indicate the involvement of lysine side chains. The rise of these bands reflects the formation of hydrogen bonds between the terminal NH_3_^+^ groups of lysine residues and the P-O^−^ groups of the phosphate sugar backbones. The increase in intensities of the bands in the amide I and II region that are overlapping with the C = O stretching region of the nucleotide bases, are indicative for structural changes of the apoprotein as well as changes in the hydrogen bonded network itself. The structural changes probably include the partially unfolding of the J_α_-helix which was suggested to be involved in the internal signal transduction as concluded from previous FTIR studies [23]. Furthermore, a similar reaction mechanism as in EL222 might be valid for aureochrome 1 as well. EL222 consists of a LOV domain that is coupled to an N-terminal HTH domain. In the dark state the DNA binding domain is bound to the β-sheet of the LOV domain [39]. This surface is directly interacting with the FMN chromophore and, consequently, illumination releases the HTH domain which is followed by dimerization and DNA binding. Aureochrome 1 might react in similar way, although the crystal structure of the LOV domain shows that the J_α_-helix is attached loosely to the β-sheet surface [13].

**Figure 7:**
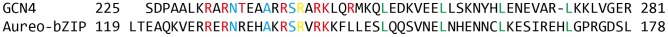
Sequence alignment of aureochrome 1 with GCN4. Sequence alignment of the bZIP domains of aureochrome 1 from *Vaucheria frigida* and of GCN4 from *S. cerevisiae*. The red marked amino acids are involved in the binding of the protein to the ribose phosphate backbone of the target DNA. The blue marked residues interact with the nucleobases [14]. The yellow marked arginines interact with both. The sequences show the typical N-X_7_-R/K motive with the hepta-repeat of leucines (colored in green) positioned exactly nine amino acids toward the C-terminus [17].

**Figure 8:**
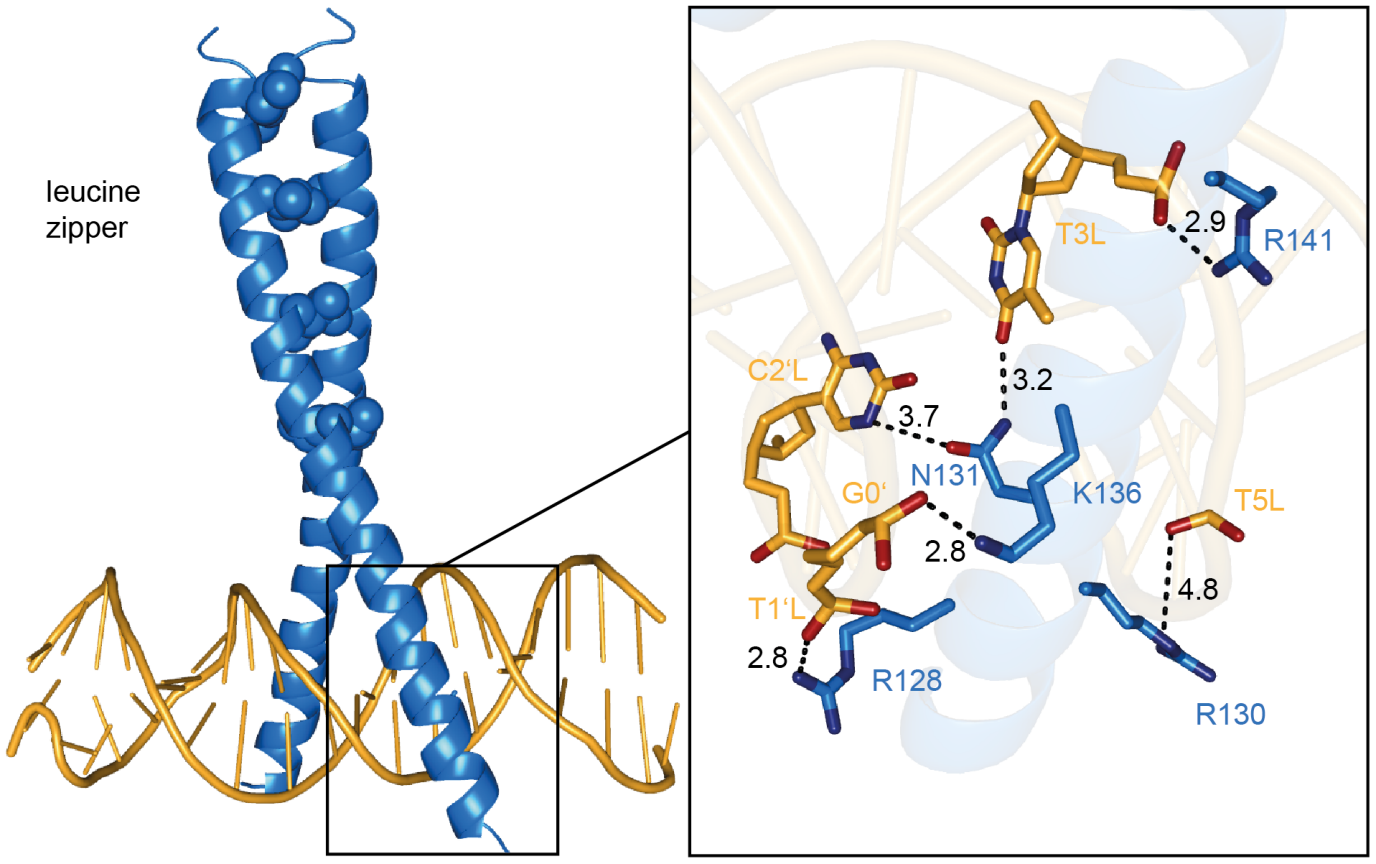
Homology model of aureochrome 1 bound to DNA. Homology model of aureochrome 1 interacting with its target DNA strand created by SWISS-Model [40], [41]. The structure was modeled on the basis of the GCN4 crystal structure (PDB:1YSA [14]). Left: Overall structure of the bZIP domain (blue) with the leucine zipper shown as spheres and the DNA as sticks (orange). The LOV domain is connected to the bZIP domain downstream of the basic region. Right: Zoom into the basic region. The image represents a rotated view on the binding complex. Protein and DNA backbones are shown as transparent cartoons. The lysine and asparagines that interact with the backbone and nucleobases of the DNA are shown as sticks and are labeled. For the bases C2'L and T3L the whole nucleotide is drawn as sticks, while for T1'L, G0′ and T5L only the phosphates of the backbone are shown. The dashed lines represent hydrogen bonds with the corresponding distances in Å.

Aureochrome 1 shares high homology with the general control protein (GCN4) from *S. cerevisiae* (PDB entry: 1YSA [14]) not only in its primary sequence (Fig. 7) but also in the sequences of the respective target DNA which is ATGACTCAT for GCN4 and TGACGT for aureochrome 1, respectively. Thus, the homology model of aureochrome 1 is meaningful and allows for a structural interpretation of the FTIR data. Figure 8 (right panel) shows that three arginine residues are involved in the interaction with the target DNA: R128 forms a hydrogen bond to the phosphate of T1'L, R141 to the phosphate of T3L and R130 to T5L. Although the distance of 4.8 Å might be too long for a hydrogen bond, K136 is interacting with the phosphate group of G0′. Besides this backbone interaction, the important N131 is hydrogen bonded to the bases C2'L and T3L. This interaction is reflected by the increased intensity of the band at 1655 cm^−1^ in the FTIR difference spectrum.

In conclusion, the FTIR experiments elucidated the interactions between the bZIP domain of full-length aureochrome 1 and its target DNA during illumination. Specific residues that are involved in the binding complex were identified by their vibrational marker bands. Subsequent studies to address the crucial role of these amino acid residues will help elucidating the mechanism of this potential optogenetic tool.
